# Prognostic Significance of KIF-12 Functioning as a Tumour Suppressor in Papillary Thyroid Carcinoma

**DOI:** 10.7150/jca.92656

**Published:** 2024-02-25

**Authors:** Luying Gao, Ruifeng Liu, Yu Xia, Aonan Pan, Xinlong Shi, Liyuan Ma, Jiang Ji, Ya Hu, Xiaoyi Li, Yuang An, Nengwen Luo, Zhiyong Liang, Liangrui Zhou, Yuxin Jiang

**Affiliations:** 1 Department of Ultrasound, State Key Laboratory of Complex Severe and Rare Diseases, Peking Union Medical College Hospital, Chinese Academy of Medical Sciences and Peking Union Medical College, Beijing, China.; 2 Department of General Surgery, State Key Laboratory of Complex Severe and Rare Diseases, Peking Union Medical College Hospital, Chinese Academy of Medical Sciences and Peking Union Medical College, Beijing, China.; 3 Department of Pathology, State Key Laboratory of Complex Severe and Rare Diseases, Peking Union Medical College Hospital, Chinese Academy of Medical Sciences and Peking Union Medical College, Beijing, China.

**Keywords:** KIF-12, Prognosis, Differentiated thyroid carcinoma, Thyroid

## Abstract

**Objective:** To explore the potential value of a novel marker, KIF-12, in the progression and prognosis of papillary thyroid carcinoma (PTC) through integrative bioinformatics analysis, and clinical sample validation of the prognostic value of KIF-12.

**Materials and Methods:** We extracted the clinicopathological data of 502 PTC patients from The Cancer Genome Atlas-Thyroid Cancer (TCGA-THCA) dataset to identify reliable differentially expressed genes (DEGs) between high and low KIF12 expression groups. Functional enrichment analysis was performed on upregulated DEGs. Gene set enrichment analysis (GESA) was performed to identify the biological pathways. We further applied Cox analysis to determine independent risk factors associated with the PTC progression-free interval (PFI), and a nomogram was established to predict disease outcome. Finally, the prognostic value of KIF12 was validated by means of clinical samples from PTC patients with and without lateral lymph node metastasis.

**Results:** On the basis of the TCGA-THCA database, we found that low KIF-12 expression was significantly related to a higher TNM stage (p<0.05), BRAF mutation status (p = 0.019), and extrathyroidal extension (p<0.001). KIF-12 was an independent prognostic factor of PTC (OR=0.319, 95% CI=0.130-0.784, P=0.013). The prognostic value of KIF12 was also successfully validated in clinical samples from twenty-nine PTC patients with lateral lymph node metastasis by comparison with twenty-two PTC patients without lymph node metastasis (P = 0.004).

**Conclusions:** We report that KIF-12 has a tumor suppressive function in PTC and may be a useful prognostic tool to predict patient outcomes.

## Introduction

Thyroid cancer (THCA) is the most common endocrine malignancy and ranks eleventh among the leading types of cancer worldwide 1. The incidence of THCA has been rising globally over recent decades, and the causes are multifactorial 2. Differentiated thyroid cancer (DTC), including papillary thyroid carcinoma (PTC) and follicular thyroid carcinoma (FTC), are the two main types of THCA, and they have relatively good prognoses. Approximately 50% of the rise in THCA is attributed to the greater detection of small PTC, especially papillary thyroid microcarcinoma (PTMC) with a maximum diameter ≤ 1 cm. However, with the increasing incidence of THCA, thyroid cancer-related mortality rates have not surged simultaneously, and this has motivated discussions regarding the overdiagnosis of THCA 3.

This is because current diagnostic techniques (ultrasound, computed tomography, and magnetic resonance imaging) are accessed on a massive scale, resulting in more detection of THCA at the early stage. Given the indolent behaviour and > 99% 10-year disease-specific survival rate of patients with low-risk PTMC 4, active surveillance (AS) instead of immediate surgery has been proposed as an alternative treatment in recent years 5. AS benefits patients in several ways; for example, postoperative complications and lifelong medication are avoided by AS 6. Nonetheless, the application of AS is based on the accurate assessment of low-risk and high-risk PTMC preoperatively, thus ensuring that high-risk patients receive surgery and additional therapeutics in a timely manner while low-risk patients are provided with more options to avoid overtreatment. Several popular guidelines, namely, the 2015 American Thyroid Association (ATA) guidelines and the Thyroid Imaging Reporting and Data System (TI-RADS), proposed by KWAK and ACR, have recommended a staging system to assess the malignancy risk of thyroid nodules preoperatively using neck ultrasound examination. Surgery and additional therapeutics are recommended for DTC with growth or aggressive behaviour. But, none of them specifically mentions how to evaluate the growth rate or aggressive behaviour of thyroid carcinoma prior to surgery 7. Therefore, identifying reliable biomarkers for predicting the biological behaviour of THCA is clinically crucial.

Previous studies have demonstrated gene signatures predicting thyroid cancer prognosis 8-10. However, these studies lack further exploration of certain genes. Advanced analysis of public databases could reveal more succinct and practical gene models to indicate the progression risk of PTC. Moreover, correlations with clinical pathological parameters and immune-related interactions will be elucidated, thus guiding individualized management. In this study, we aimed to analyse thyroid cancer datasets from the public databases to identify reliable differentially expressed genes (DEGs) in PTCs and identify DEGs associated with the progression-free interval (PFI) of PTC. We selected a potential gene KIF-12 predicting progression behavior of THCA using gene expression and clinical data from the TCGA-THCA dataset, and validated in our clinical samples. KIF12 is an orphan kinesin-12 family motor protein, which consists of microtubule-associated motor proteins involved in organizing the cytoskeleton and intracellular transport 11. However, the value of KIF12 in tumors has never been reported.

## Materials and Methods

### Data acquisition and fundamental processing

Harmonized gene expression data (HTSeq-FPKM and HTSeq counts) and associated clinicopathological information of thyroid carcinoma were downloaded from the TCGA database (https://portal.gdc.cancer.gov/). The inclusion criteria of subjects were as follows: (i) histologically confirmed PTC, (ii) age>18 years old. Cases (i) without clinical information were excluded. There were 502 samples with gene transcriptome data retained for further analysis (58 normal and 502 tumour). After transforming level 3 HTSeq-FPKM into the format of transcripts per million reads (TPM), the expression level of KIF12 between tumour and normal samples was compared. Furthermore, differentially expressed genes (DEGs) between the high and low KIF12 expression THCA groups were identified using the DESeq2 package in R. The cut-off criteria were |log2-fold change (FC)| >1 and P< 0.05 12.

### Enrichment and interaction analysis

Functional enrichment analysis comprising gene ontology (GO) analysis and Kyoto Encyclopedia of Genes and Genomes (KEGG) pathway analysis was performed on upregulated DEGs using the clusterProfiler package. Gene set enrichment analysis (GSEA) was achieved by the clusterProfiler package in R to determine whether an a priori defined set of genes differs significantly between high- and low- KIF12 expression subsets 13. The enriched pathways based on the varied expression level of KIF12 were classified by the adjusted p value (<0.05), false discovery rate (FDR) q-value (<0.25) and normalized enrichment score (|NES|>1) 14. The immune infiltration analysis of THCA was conducted by the single-sample gene set enrichment analysis (ssGSEA) method using the GSVA package. Twenty-four types of immunocytes with signature genes labelled in the literature were correlated with KIF12 expression in all THCA samples 15. To exhibit the interaction among the DEGs, protein‒protein interaction (PPI) networks were constructed employing the Search Tool for the Retrieval of Interacting Genes (STRING) (http:string-db.org/) database 16. An interaction combined score>0.7 was set to explore densely connected network elements using the Molecular Complex Detection (MCODE) algorithm 17. This study satisfies the publication requirements stated by TCGA (http://cancergenome.nih.gov/publications/publicationguidelines).

### Validation in clinical samples

We retrospectively reviewed the medical records of PTC patients who underwent thyroid surgery at our centre between August 2021 and October 2021. The inclusion criteria of subjects were as follows: (i) thyroidectomy performed together with central and, when necessary, therapeutic lateral neck dissection, (ii) histologically confirmed PTC, (iii) target lesion size of r ≤1 cm (iv) age>18 years old. The exclusion criteria: (i) previous thyroid surgery or related invasive operation for the treatment of target lesions. Thirty PTC patients with histologically confirmed lateral lymph node metastases were included in the experimental group. Thirty PTC patients without evidence of lymph node metastases were enrolled in the control group. Pathological tissues of PTC patients meeting the inclusion criteria were collected, and tissue sections and paraffin blocks were produced. RNA was extracted from tissues from patients using an RNeasy FFPE Kit (RNeasy FFPE Kit, QIAGEN, Hilden, Germany). cDNA was then synthesized using a reverse transcriptase system (High-Capacity cDNA Reverse Transcription Kit, Thermo Fisher Scientific, Waltham, USA). RT‒qPCR was used to validate gene expression in accordance with the manufacturer's instructions (SYBR™ Green PCR Master Mix, Thermo Fisher Scientific, Waltham, USA). Relative gene expression was calculated using the 2-ΔΔCT method. Primers for RT‒qPCR were purchased from Sangon Biotech (Shanghai, China). Finally, there were twenty-nine patients in the experimental group and twenty-two patients in the control group.

### Statistical analysis

We used R (v.3.5.1) and SPSS software version 19.0 (IBM, Armonk, NY, USA) to complete the statistical analysis. The Wilcoxon rank-sum test was used for comparisons of KIF12 expression levels between THCA and normal samples. Clinically, the normality of variables was verified by the Shapiro‒Wilk test. For parametric data, an unpaired t test was used to evaluate differences between the two groups. For nonparametric data, differences between groups were analysed using a Mann‒Whitney U test. The progression-free interval (PFI) was defined as the period from the date of diagnosis until the date of the newly discovered tumour event, mainly including locoregional recurrence, distant metastasis or progression of the disease in our selected THCA samples. The chi-square test was applied to analyse categorical variables, and the Kruskal‒Wallis test was used for comparisons of continuous variables. Multivariate Cox regression analyses and the Kaplan‒Meier method were adopted to identify independent prognostic factors. Moreover, a nomogram was developed according to the results of the multivariate analysis for visualizing the PFI probability at 5 years individually. The calibration curves were also created by plotting predicted probabilities against the observed rates. A 2-sided P<0.05 was viewed as the threshold for a statistically significant difference between the low- and high-expression KIF12 groups.

## Results

### Association of KIF-12 expression with clinical characteristics

To investigate whether KIF-12 expression is related to the clinical parameters of PTC, variables such as sex, age, race, neoplasm location, focus number, thyroid gland disorder history, extrathyroidal extension, BRAF status, RAS status, TNM stage, pathologic stage, residual tumour and histologic type were analysed. As shown in Table 1, the results of the analyses showed that there were significant differences in T stage (p = 0.001), N stage (p = 0.03), pathologic stage (p = 0.003), residual tumour (p = 0.023), histologic type (p = 0.003), race (p = 0.025), extrathyroidal extension (p<0.001), and BRAF status (p = 0.019) in the TCGA cohort Table 1.

### Prognostic value of KIF-12 in d-THCA

**Figure 1:**
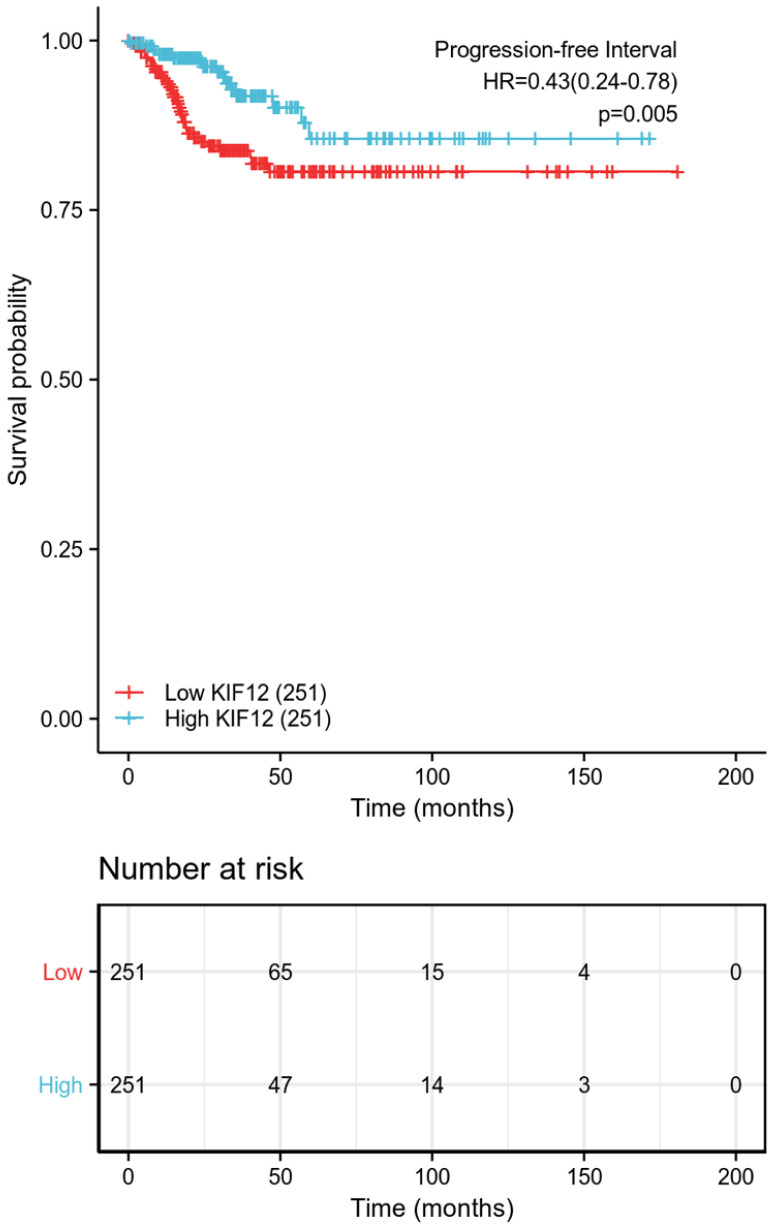
Progression-free survival between the high-low expression groups of KIF-12.

**Figure 2:**
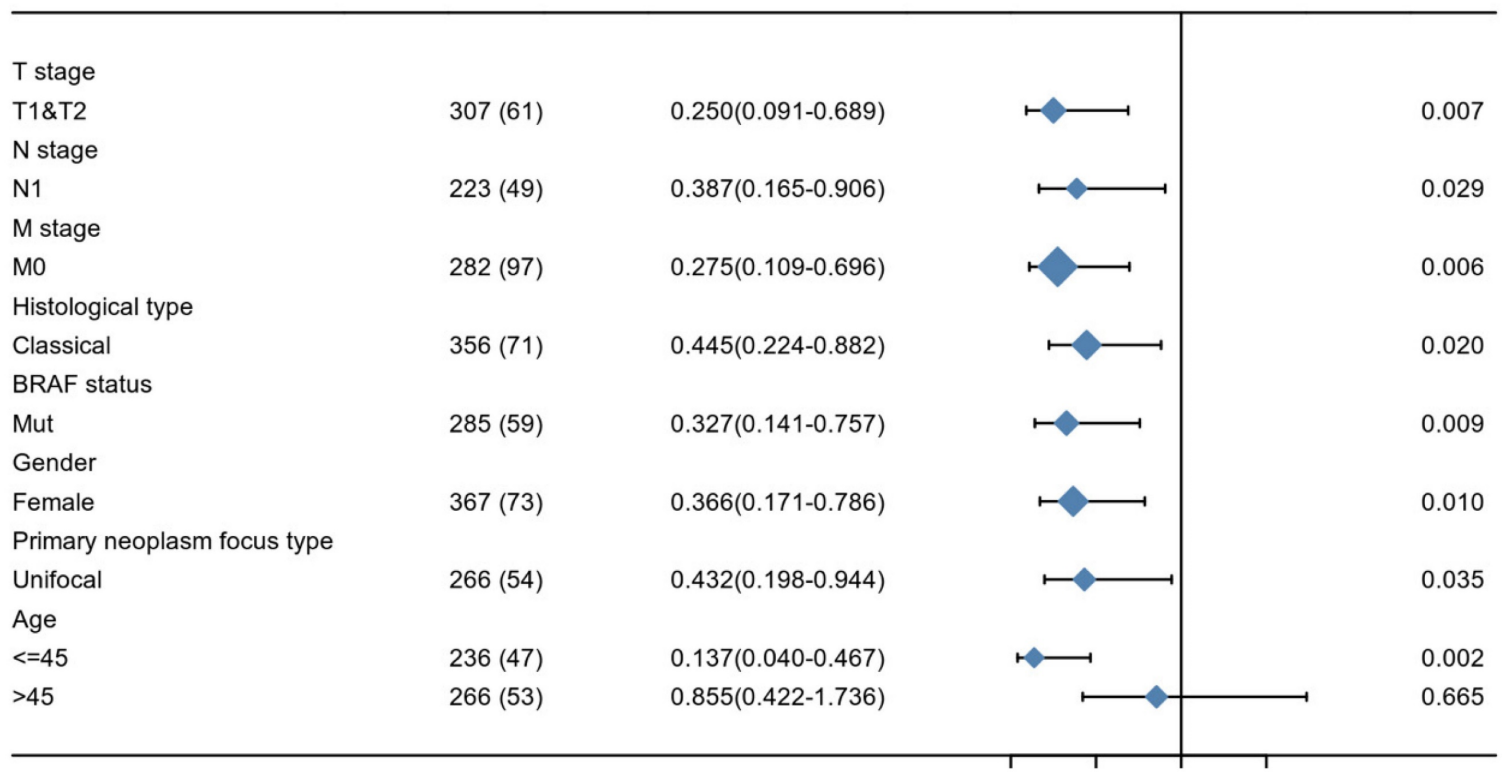
Subgroup analysis of KIF-12 expression.

The relationship between KIF-12 expression as well as clinicopathological factors and progression-free interval was further explored by COX regression analysis. In the univariate Cox regression analysis, T stage, M stage, pathologic stage, extrathyroidal extension, and KIF-12 expression satisfied the threshold of p<0.1 and were included in the multivariate study. Multivariate Cox analysis revealed that M stage (OR=6.818, 95% CI=2.103-22.106, p=0.001) and KIF-12 expression (OR=0.319, 95% CI=0.130-0.784, P=0.013) were independent prognostic factors for PFI [Table 2], and the results were demonstrated in the form of a nomogram for predicting the individual probability of PFI [Figure 1]. In terms of subgroup analysis, KIF-12 showed significant expression differences in the T1 and T2 tumour stage subsets, M0 and N1 stage subsets, classical PTC, female population, BRAF mutation group, patients with unifocal neoplasm and patients aged ≤ 45 [Figure 2].

### Identification of DEGs in differentiated thyroid carcinoma

**Figure 3:**
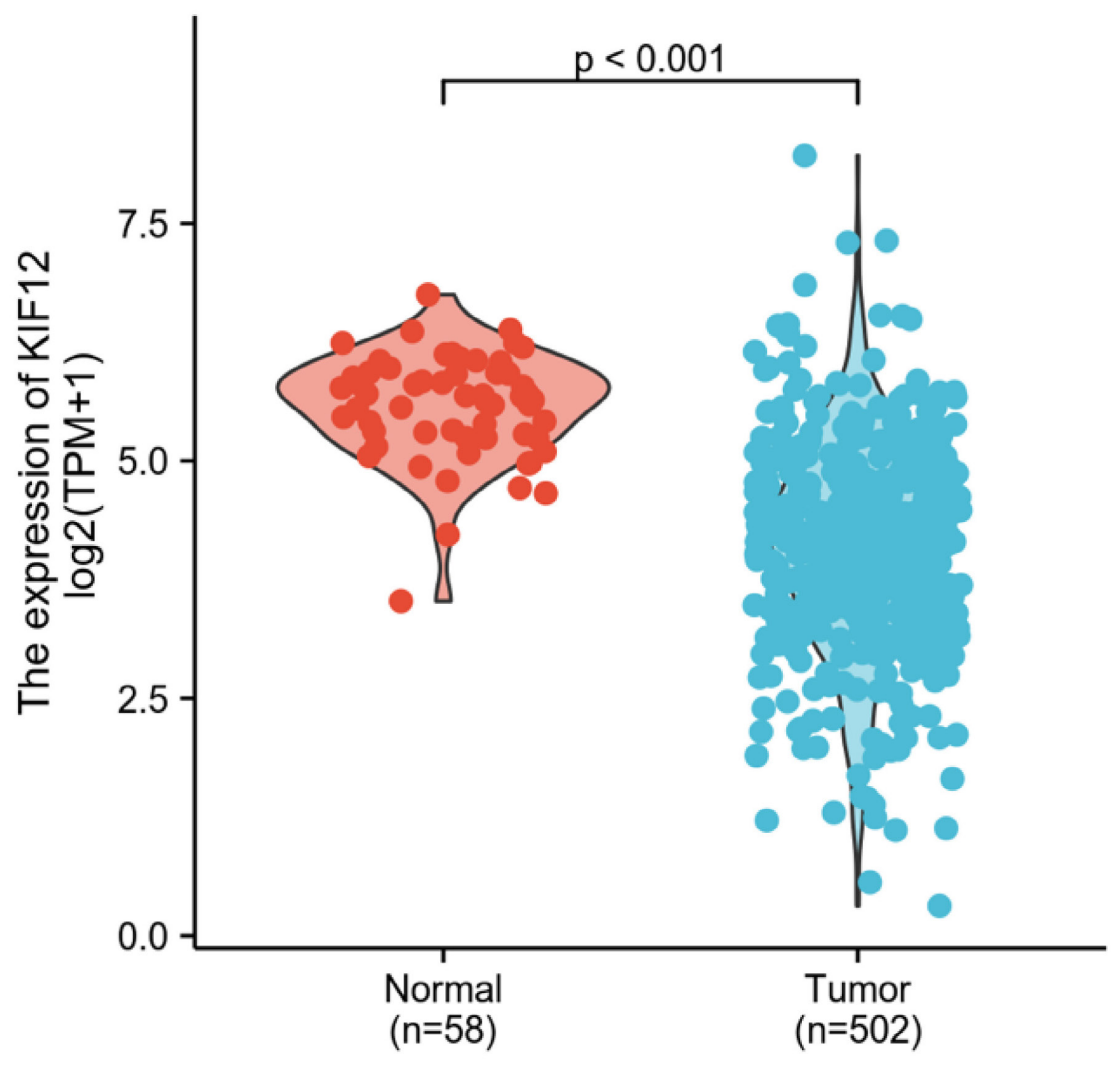
Comparison of KIF-12 expression in tumour specimens and normal specimens.

**Figure 4:**
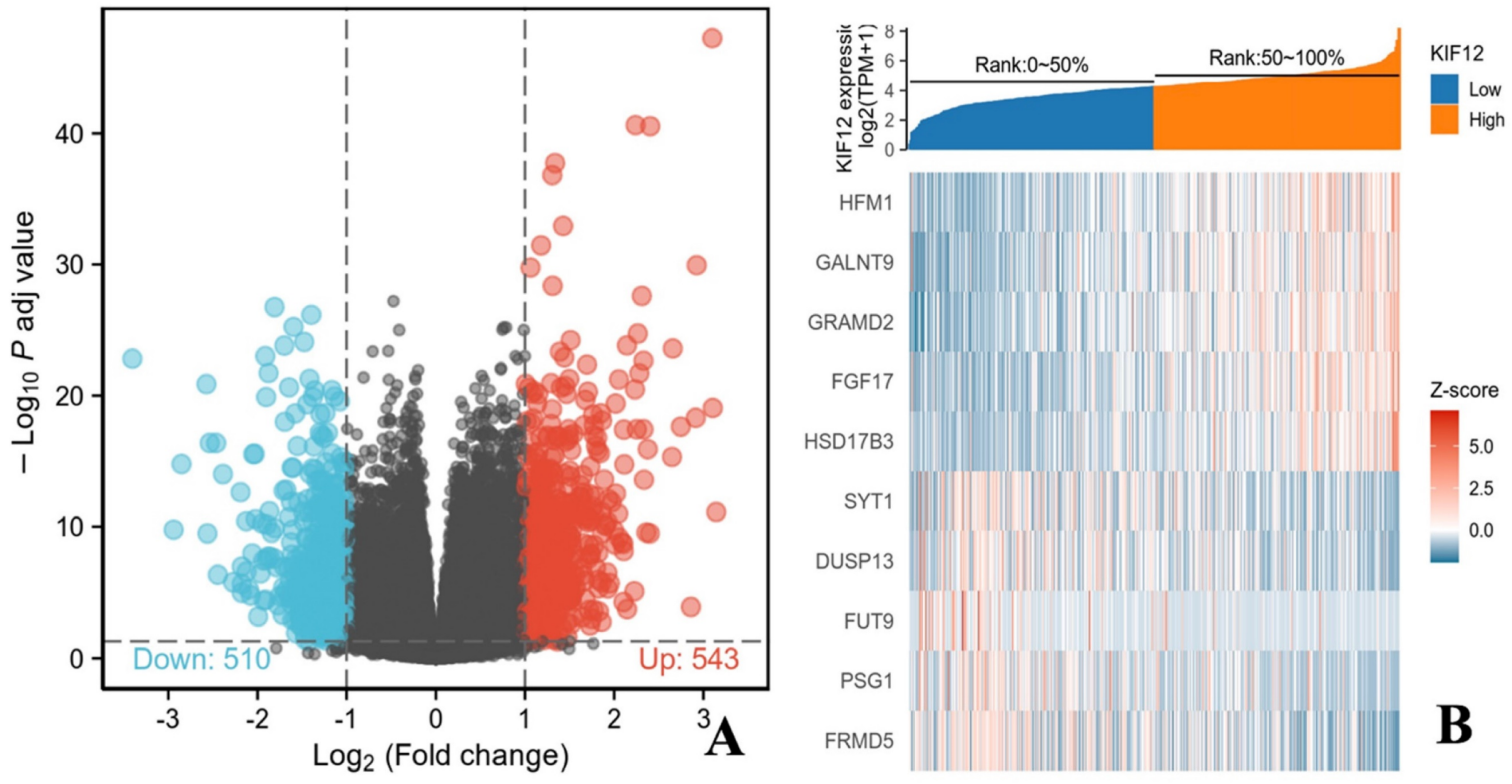
Genes related to KIF-12 in thyroid cancer. (A). Volcano plot showing KIF-12 and associated genes' expression. Dark red dots represent genes positively correlated with KIF-12 and dark green dots represent genes negatively correlated with KIF-12. (B). Heat map showing top genes correlated with KIF-12.

In total, 502 tumour samples and 58 adjacent noncancerous samples were obtained from PTC patients in the TCGA cohort with matched RNA-sequencing data and clinical information. As shown in Figure 3, KIF-12 expression was significantly lower in tumour specimens than in normal control specimens. This suggests the potential role of KIF-12 as a tumour-suppressing gene in PTC. The comparison of the high- and low-KIF-12 expression groups generated 1054 DEGs in total (543 upregulated and 510 downregulated) for further analysis using the cut-off criteria of padj < 0.05 and |logFC| > 1 [Figures 4].

### Predicted KIF-12 functions and pathways based on DEGs

**Figure 5:**
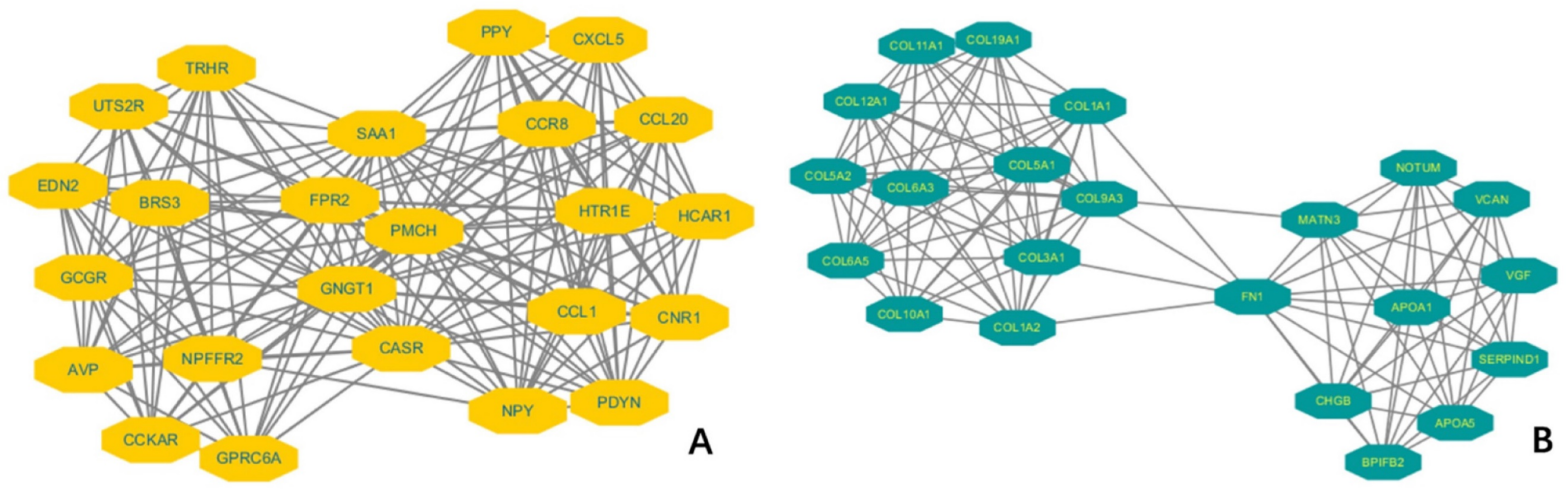
Two most significant molecular complex detection (MCODE) components of a protein‒protein interaction (PPI) regulatory network in thyroid cancer patients. (A). MCODE 1. (B). MCODE 2.

To understand the molecular mechanism of KIF-12 in d-THCA, gene ontology (GO) and Kyoto Encyclopedia of Genes and Genomes (KEGG) functional enrichment analysis were performed on the DEGS. The GO analysis categorized the genes into three domains, namely, biological process, cellular component and molecular function. Regarding biological process, a variety of processes, including extracellular structure organization (p = 1.90E-12), extracellular matrix organization (p = 6.37E-12), and collagen fibril organization (p = 4.10E-06), were shown to be involved. Regarding cellular component, collagen-containing extracellular matrix (p = 7.91E-15) and collagen trimer (p = 1.19E-13) were mostly indicated. Regarding molecular function, extracellular matrix structural constituent (p = 9.37E-11), receptor ligand activity (p = 7.41E-10) and extracellular matrix structural constituent conferring tensile strength (p = 2.67E-08) were involved. Moreover, in the GSEA, the gene signatures were shown to be linked to the metabolic functions of amine-derived hormones, the TFF pathway and transporter disorders. Moreover, KEGG analysis suggested that the DEGs pointed to pathways involving protein digestion and absorption (p = 1.50E-05) and neuroactive ligand‒receptor interaction (p = 1.1E-03). However, regarding immune infiltration, KIF-12 showed a low correlation coefficient with different types of immune cells, such as Th1, Th2, Th17, Treg cells, NK cells, B cells, aDC cells, cytotoxic cells, and macrophages. Among these immune cells, Th2 cells exhibited the highest inverse correlation with KIF-12 expression levels (Spearman r = 0.358). Furthermore, we constructed a protein‒protein interaction (PPI) regulatory network using the String database to elucidate the protein‒protein interaction in d-THCA patients and presented the two most significant molecular complex detection (MCODE) components [Figures 5].

### Verification in clinical samples

Postoperative histopathologic results showed that all lesions were papillary thyroid carcinoma. There were 69% female with lateral lymph node metastases (LLNM) and 72.3% female patients without lymph node metastases (LNM). The sizes of the nodules in the patients with LLNM and patients without LNM were 0.60 ± 0.24 cm and 0.54 ± 0.25 cm, respectively. There were no significant differences in sex or tumour size (P = 0.51; P = 0.41). The average age of patients with LLNM 34.5 ± 8.3 years old, and patients without LNM were 43.8 ± 9.7 years old (P=0.001) [Table 3].

**Figure 6:**
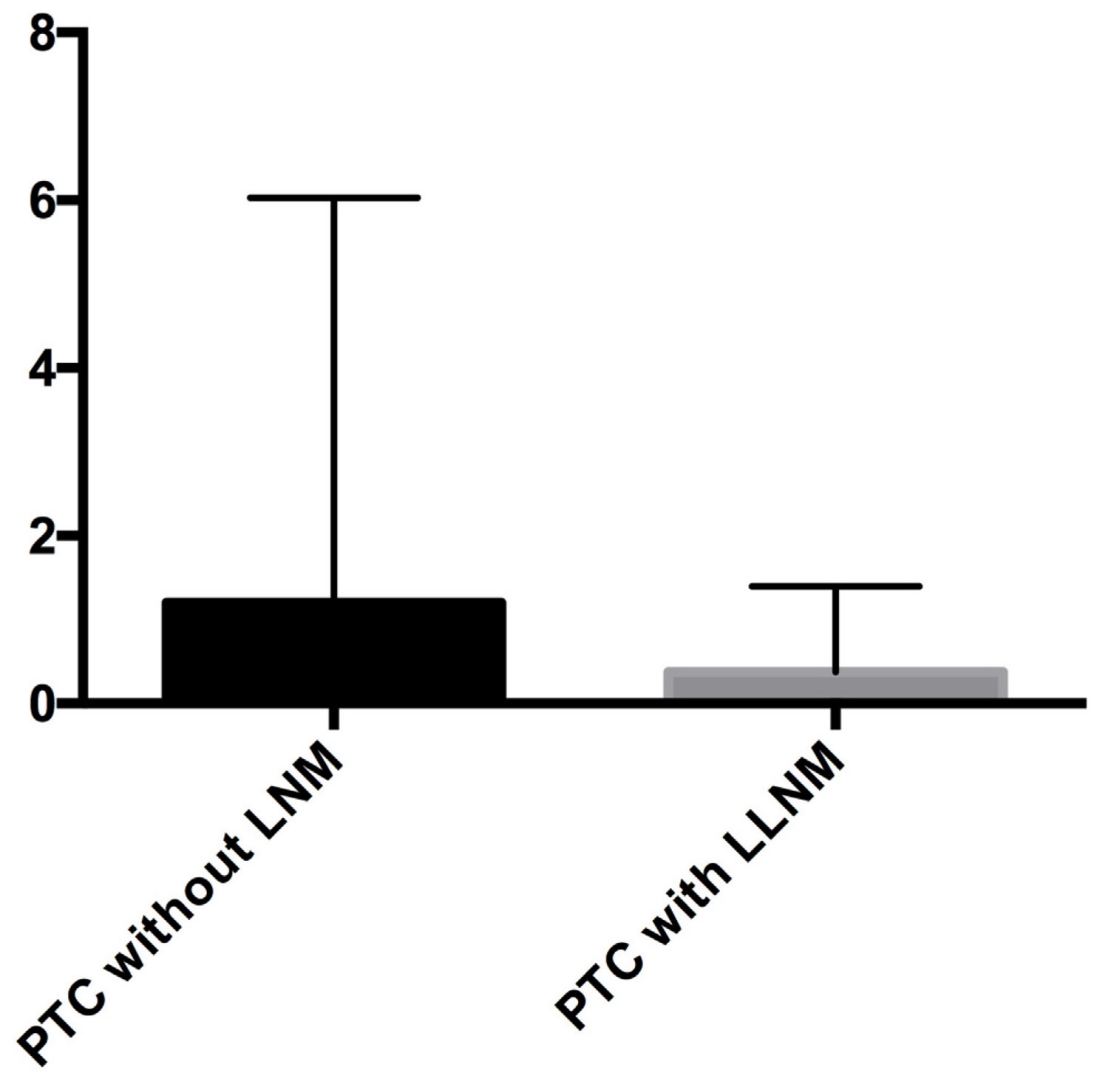
RT‒PCR verification in clinical samples by comparison the patients with lateral lymph node metastases (LLNM) and without lymph node metastases (LNM).

Pathological tissues were collected from PTC patients, and the KIF-12 expression of was verified using quantitative reverse transcription polymerase chain reaction (RT‒qPCR). The results showed that compared with that in the PTC samples from patients without LNM, the expression of KIF-12 in PTC samples from patients with LLNM was significantly lower (P = 0.004) (median: 0.38 vs 1.20) [Figures 6]. Our results indicated that low KIF-12 expression was related to LLNM in PTC.

## Discussion

Although the increasing incidence of PTC has been widely reported, AS management has been considered an alternative to immediate surgery for patients with PTMC. However, some patients with PTMC have poor clinical features, such as clinically significant regional lymph nodes (LNs). However, it is not yet clear how to predict the aggressive behaviour of PTC prior to surgery. Therefore, identifying reliable biomarkers to predict the biological behaviour of PTC is clinically crucial. In this study, we explored the potential role of KIF-12 in the progression and prognosis of PTC. We found that low PTC expression was significantly related to PTC progression. Moreover, KIF-12 was markedly decreased in PTC patients with lateral lymph node metastasis (LLNM).

The role of KIF-12 in tumours has seldom been reported. On the basis of data in the TCGA database, we found the potential role of KIF-12 as a tumour suppressor gene in differentiated thyroid carcinoma, and KIF-12 expression was an independent prognostic factor for thyroid cancer. The results showed that the downregulation of KIF-12 expression enhances the progression of differentiated thyroid carcinoma, and the effect may be more significant among younger female patients with unifocal PTC showing lymph node metastasis and BRAF mutation. The results were also successfully validated in clinical samples from PTC patients with LLNM. The kinesin superfamily (KIFs) is a class of molecular motors that mainly mediates intracellular molecular transport. Kinesins are motor proteins that transport cargoes by walking unidirectionally along microtubule tracks, hydrolyzing one molecule of ATP at each step. Abnormal expression of genes in the KIF protein family in tumours may alter the distribution of genetic material, affect spindle formation, and cause defects in cell division, thereby mediating tumour development. In addition, kinesins are key participants in chromosomal and spindle movements during mitosis. KIF12 is an orphan kinesin-12 family motor protein 11. KIF12 is an orphan kinesin-12 family motor protein, which consists of microtubule-associated motor proteins involved in organizing the cytoskeleton and intracellular transport 18. In addition, KIF12 has been shown to be required for normal mitosis, Myosin II localization in the cleavage furrow, and cytokinesis in Dictyostelium 19. KIF12 has previously been reported to be involved in the pathogenesis of polycystic kidney disease as a modifier gene 20, affecting insulin secretion from β-cells and thus mediating the progression of diabetes mellitus 21. Previous cases described pathogenic KIF12 variants as a potential cause for cholestatic liver disease 22. Its function in tumours has not been reported, and only a part of the KIF12 function is understood. This is the first study to report KIF-12 as a prognostic factor for thyroid cancer.

Furthermore, to understand the molecular mechanism of KIF-12 in thyroid cancer, GO and KEGG functional enrichment analysis were performed on the DEGs. The molecular function terms included extracellular matrix structural constituent, receptor ligand activity and extracellular matrix structural constituent conferring tensile strength. GSEA was conducted to better understand the potential function of KIF-12 in thyroid cancer. GSEA demonstrated that gene signatures were linked to the metabolic functions of amine-derived hormones, the TFF pathway and transporter disorders, which may provide some hints for interactions of KIF-12 on thyroid cancer development and progression.

Although the prognostic value of KIF-12 in thyroid cancer has been demonstrated in our study for the first time, there were still some limitations in our work. The data in the TCGA database and clinical cases are limited, and access to more public data and patient data will be helpful toward strengthening our results. Second, the underlying mechanisms regarding KIF-12 actions are not well investigated, further mechanistic study to assess the impact of KIF-12 on thyroid cancer are needed.

In conclusion, our study explored the prognostic role of KIF-12 in thyroid cancer for the first time. On the basis of integrated bioinformatics analyses of data in TCGA database, we also conducted further validation experiments in clinical cases to further confirm that low KIF-12 expression was correlated with the progression and prognosis of thyroid cancer. Collectively, our findings indicate that KIF-12 is a promising prognostic suppressor of thyroid cancer.

## Figures and Tables

**Table 1:** Association of KIF-12 expression with clinical characteristics

Characteristics	Level	Low expression of KIF12	High expression of KIF12	p	test
n		251	251		
T stage (%)	T1	57(22.8%)	86(34.4%)	0.001	exact
	T2	77(30.8%)	87(34.8%)		
	T3	99(39.6%)	71(28.4%)		
	T4	17(6.8%)	6(2.4%)		
N stage (%)	N0	105(45.5%)	124(56.1%)	0.030	
	N1	126(54.5%)	97(43.9%)		
M stage (%)	M0	128(97.7%)	154(96.2%)	0.521	exact
	M1	3(2.3%)	6(3.8%)		
Pathologic stage (%)	Stage I	124(49.4%)	157(63.1%)	0.003	
	Stage II	26(10.4%)	26(10.4%)		
	Stage III	63(25.1%)	49(19.7%)		
	Stage IV	38(15.1%)	17(6.8%)		
Residual tumour (%)	R0	186(83.8%)	198(90.8%)	0.023	exact
	R1	32(14.4%)	20(9.2%)		
	R2	4(1.8%)	0(0.0%)		
Histological type (%)	Classical	175(69.7%)	181(72.1%)	0.003	exact
	Follicular	45(17.9%)	56(22.3%)		
	Other	3(1.2%)	6(2.4%)		
	Tall Cell	28(11.2%)	8(3.2%)		
Sex (%)	Female	185(73.7%)	182(72.5%)	0.840	
	Male	66(26.3%)	69(27.5%)		
Race (%)	Asian	16(8.1%)	35(16.4%)	0.025	
	Black or African American	16(8.1%)	11(5.2%)		
	White	165(83.8%)	167(78.4%)		
Neoplasm location (%)	Bilateral	36(14.5%)	50(20.2%)	0.117	
	Isthmus	15(6.0%)	7(2.8%)		
	Left lobe	85(34.3%)	90(36.3%)		
	Right lobe	112(45.2%)	101(40.7%)		
Primary neoplasm focus type (%)	Multifocal	113(45.6%)	113(46.3%)	0.940	
	Unifocal	135(54.4%)	131(53.7%)		
Thyroid gland disorder history (%)	Lymphocytic Thyroiditis	40(18.0%)	31(14.0%)	0.260	
	Nodular Hyperplasia	34(15.3%)	34(15.3%)		
	Normal	132(59.5%)	148(66.7%)		
	Other, specify	16(7.2%)	9(4.1%)		
Extrathyroidal extension (%)	No	145(59.4%)	186(77.5%)	<0.001	
	Yes	99(40.6%)	54(22.5%)		
BRAF status (%)	Mut	156(64.2%)	129(53.3%)	0.019	
	WT	87(35.8%)	113(46.7%)		
NRAS status (%)	Mut	22(9.1%)	17(7.0%)	0.513	
	WT	221(90.9%)	225(93.0%)		
HRAS status (%)	Mut	10(4.1%)	7(2.9%)	0.623	exact
	WT	233(95.9%)	235(97.1%)		
Age (median [IQR])		49.00[35.00,61.00]	44.00[35.00,55.50]	0.060	nonnorm

**Table 2:** Prognostic value of KIF-12 in d-THCA

Characteristics	Total (N)	HR (95% CI) Univariate analysis	P value Univariate analysis	HR (95% CI) Multivariate analysis	P value Multivariate analysis
T stage (T3&T4 vs. T1&T2)	500	2.507(1.434-4.385)	0.001	0.752(0.181-3.115)	0.694
N stage (N1 vs. N0)	452	1.578(0.885-2.814)	0.122		
M stage (M1 vs. M0)	291	7.542(2.860-19.891)	<0.001	6.818(2.103-22.106)	0.001
Pathologic stage (Stage III & Stage IV vs. Stage I & Stage II)	500	2.615(1.515-4.515)	<0.001	2.712(0.811-9.068)	0.105
Residual tumour (R1&R2 vs. R0)	440	1.773(0.856-3.674)	0.123		
Histological type (Classical vs. Follicular & Other & Tall Cell)	502	1.022(0.553-1.888)	0.944		
Age (>45 vs. <=45)	502	1.618(0.924-2.834)	0.092	1.109(0.370-3.323)	0.853
Sex (Male vs. Female)	502	1.529(0.863-2.709)	0.146		
Race (White vs. Asian & Black or African American)	410	1.183(0.529-2.646)	0.682		
Neoplasm location (Bilateral vs. Isthmus & Left lobe & Right lobe)	496	1.150(0.558-2.370)	0.704		
Primary neoplasm focus type (Multifocal vs. Unifocal)	492	1.036(0.595-1.802)	0.901		
Thyroid gland disorder history (Lymphocytic Thyroiditis & Nodular Hyperplasia & Other, specify vs. Normal)	444	0.966(0.520-1.795)	0.913		
Extrathyroidal extension (Yes vs. No)	484	1.995(1.151-3.458)	0.014	1.054(0.303-3.663)	0.935
BRAF status (Mut vs. WT)	485	1.266(0.703-2.280)	0.432		
NRAS status (Mut vs. WT)	485	1.958(0.830-4.615)	0.125		
HRAS status (Mut vs. WT)	485	1.033(0.251-4.258)	0.964		
KIF12 (High vs. Low)	502	0.431(0.239-0.777)	0.005	0.319(0.130-0.784)	0.013

**Table 3:** Characteristics of clinical samples

	Lateral lymph node metastasis+	Lymph node metastasis -	P value
No.	29	22	
Age, years (mean)	34.5	43.8	0.001
Sex (n, %)			0.51
Male	9 (31%)	6 (27.3%)	
Female	22 (69%)	16 (72.7%)	
Tumour size (cm)	0.60	0.54	0.41

## Abbreviations

PTC: Papillary thyroid carcinoma
TCGA-THCA: Cancer Genome Atlas-Thyroid Cancer
DEGs: Differentially expressed genes
GESA: Gene set enrichment analysis
PFI: Progression-free interval
AS: Active surveillance
ATA: American Thyroid Association
TI-RADS: Thyroid Imaging Reporting and Data System
PTMC: Papillary thyroid microcarcinoma
TPM: Transcripts per million reads
GO: Gene ontology
KEGG: Kyoto Encyclopedia of Genes and Genomes
FDR: False discovery rate
ssGSEA: Single-sample gene set enrichment analysis
MCODE: Molecular Complex Detection
PPI: Protein‒protein interaction
STRING: Search Tool for the Retrieval of Interacting Genes
LLNM: Lateral lymph node metastases
LNM: Lymph node metastases
LNs: Lymph nodes
KIFs: Kinesin superfamily
